# Granulation tissue-like spindle cell (sarcomatoid) carcinoma of the head and neck: a deceptively bland-looking underdiagnosed malignancy

**DOI:** 10.1007/s00428-024-03770-3

**Published:** 2024-02-26

**Authors:** Alessandro Franchi, Abbas Agaimy

**Affiliations:** 1 Section of Anatomic Pathology, Department of Translational Research, University of Pisa, 56124 Pisa, Italy; 2 Institute of Pathology, University Hospital Erlangen, Friedrich‐Alexander University Erlangen‐Nürnberg (FAU), Comprehensive Cancer Center (CCC) Erlangen-EMN, 91054 Erlangen, Germany

**Keywords:** Spindle cell squamous cell carcinoma, Sarcomatoid carcinoma, Granulation tissue, Head and neck, Immunohistochemistry

## Abstract

The diagnosis of head and neck spindle cell squamous carcinoma (SC-SCC) is often challenging. Lesions with a prominent inflammatory infiltrate and reactive vessels may have a granulation tissue-like appearance, therefore being difficult to distinguish from reactive lesions, like contact ulcers, post-intubation granulomas, inflammatory pseudotumors, or benign vascular lesions. In this study, we analyzed the clinicopathological features of a series of 17 head and neck SC-SCC with granulation tissue-like appearance. All patients, but two, were males, ranging in age between 57 and 80 years. The larynx was the most frequently affected site (*n* = 12), followed by the tongue (*n* = 4). One tumor was hypopharyngeal. Most consult cases were submitted with benign suggestion or because of unexpected recurrences of granulation tissue polyps. Histologically, all lesions consisted of an ulcerated polypoid proliferation of moderately to markedly atypical spindle cells, with a minor component of conventional invasive or in situ squamous carcinoma. At least one cytokeratin cocktail was positive in 13 cases. The staining was limited to a few neoplastic cells in most cases. Positivity for p63, p40, and cytokeratins 5/6 was detected only in the conventional squamous cell carcinoma component, when present. ALK1 was negative in all cases. Sixteen cases were tested for p53 and all showed aberrant expression (12 diffusely positive and 4 of null-phenotype). The diagnosis of granulation tissue-like SC-SCC is challenging due to the close clinical and histological overlap with several benign conditions. Since the expression of epithelial markers is limited, the use of an immunohistochemical panel including p53 is recommended.

## Introduction

Spindle cell squamous carcinoma (SC-SCC) is defined as a biphasic tumor composed of a conventional squamous cell carcinoma component (either invasive or in situ) and a spindle cell and epithelioid/pleomorphic component with sarcomatoid appearance [1]. The oral cavity and the larynx are the most commonly affected sites in the head and neck region [2, 3].

The pathogenesis of SC-SCC has been debated for years, and it is now accepted that the tumor has a monoclonal origin, with both components originating from a single stem cell. The acquisition of a mesenchymal-like phenotype with transition from epithelial to spindle cell phenotype seems to be associated with altered expression of the cadherin-catenin complex, with a mechanism reminiscent of the epithelial-mesenchymal transition [4, 5].

The diagnosis of SC-SCC is often challenging, mainly because the conventional squamous cell carcinoma component may be limited and difficult to recognize or is lacking altogether. When the spindle cell sarcomatoid component predominates, several benign and malignant mesenchymal lesions may be entered in the differential diagnosis, according to the architecture of the lesion, the stromal characteristics, and the morphology of the predominant cellular constituent [6, 7]. Tumors with a polypoid architecture, composed by a proliferation of bland spindle cells, accompanied by a prominent inflammatory infiltrate and reactive vessels, may have a fibrovascular or granulation tissue-like appearance, therefore being very difficult to distinguish from reactive lesions, like contact ulcers, post-intubation granulomas, benign vascular tumors, and inflammatory pseudotumors.

In this study, we examined the clinicopathological features of a series of SC-SCCs with granulation tissue-like appearance, aiming to define the diagnostic criteria and to explore the utility of immunohistochemical markers.

## Materials and methods

### Case selection

After a search conducted in the files of our departments and in our consultation files, we selected 17 cases of SC-SCC of head and neck mucosal sites histologically showing a prominent inflammatory infiltrate with abundant accompanying capillary vessels. All the available histologic slides were reviewed, and key histopathologic parameters were recorded. Five specimens of laryngeal granulation tissue were selected and included for comparison.

### Immunohistochemistry

Immunohistochemical studies for cytokeratins AE1/AE3 (AE1/AE3/PCK26, Roche Diagnostics), CAM5.2 (Roche Diagnostics), and 5/6 (D5/16B4, Roche Diagnostics), as well as for p53 (DO-7, Roche Diagnostics), p63 (SSI6, 1:100; DCS), p40 (ΔNp63, polyclonal, 1:100; Zytomed), smooth muscle actin (clone 1A4, 1:400; Dako), desmin (DE-R-11, Roche Diagnostics), CD31 (JC70, Roche Diagnostics), CD34 (QBEnd/10, 1:200; Dako) and ALK1 (D5F3, 1:100; Cell Signaling) were performed on formalin-fixed paraffin embedded tumor tissue sections with BenchMark® Ultra stainer (Ventana, Tucson, AZ, USA).

Expression of p53 was recorded either as wild-type when heterogeneous nuclear staining was observed, or aberrant, including overexpression (neoplastic cells with uniformly strong nuclear staining indicating missense mutation) and complete lack of expression (null-phenotype indicating nonsense mutation) [8].

## Results

The clinicopathological features of our series are summarized in Table 1. There were 15 males and 2 females, ranging in age between 57 and 80 years (mean 69.1 years). The most commonly affected sites were the larynx (*n* = 12) and the tongue (*n* = 4). One tumor was hypopharyngeal. Clinically, most of the lesions appeared as small polyps and were interpreted as benign. Initial treatment consisted of conservative surgery in all cases. One patient with SC-SCC of the tongue had been treated for conventional SCC of the tongue 12 years before. Follow-up was available for eight patients. Three patients with laryngeal tumors experienced local recurrences, two of which were treated with multiple conservative surgeries and one with total laryngectomy followed by radiotherapy. The remaining five patients were alive with no evidence of disease after a mean of 14 months.

**Table 1:** Clinicopathological features of granulation tissue-like spindle cell carcinoma of the upper aerodigestive tract (n = 17)

No	Age/gender	Site	Size cm	Submitted diagnosis	SIL/CIS	Immunohistochemistry	TP53	Follow-up
1	63/M	Hypopharynx	NA	NOS	No	CK18, CK5/6 and p63 negative	Aberrant positive	NA
2	76/M	Right vocal cord	NA	Recurring granulation tissue polyp	CIS	CK18, CK5/6 and p63 negative	Aberrant negative	Multiple local relapses over 3 years
3	65/M	Margin of the tongue	2.5	Granulation tissue polyp vs neoplasm	No	AE1/AE3 and p63 positive	Aberrant positive	NA
4	74/M	Larynx	2.6	Inflammatory myofibroblastic tumor vs myxofibrosarcoma	No	AE1/AE3 and p63 negative	Aberrant positive	NA
5	62/M	Left vocal cord	0.3 cm biopsies	Granulation tissue polyp- vs sarcoma	No	AE1/AE3 and p63 negative	Aberrant positive	NA
6	80/M	Left vocal cord	1	Suspicious granulation tissue polyp	Minute CIS	AE1/AE3 and p63 negative	Aberrant positive	NA
7	67/M	Larynx	NA	Granulation tissue polyp vs scar tissue	No	CK18 + , AE1/AE3 and p63 negative	Aberrant positive	Multiple local relapses over 2 years
8	79/M	Tongue	NA	Suspicious granulation polyp	Minute SCC foci	AE1/AE3 positive, p63 positive in the SCC foci	Aberrant positive	SCC base of tongue 12 years before
9	67/F	Tongue	NA	Proliferative myositis	No	AE1/AE3, CK5/6 and p63 positive	Aberrant positive	NA
10	67/M	Anterior left vocal cord	0.5	Laryngeal polyp	LG SIL adjacent	AE1/AE3 and P63 negative	Aberrant negative	Alive and well at 45 months
11	65/M	Middle right vocal cord	1.2	Laryngeal polyp vs carcinoma	HG SIL adjacent	AE1/AE3 and P63 in scattered cells	Aberrant negative	Alive and well at 32 months
12	57/M	Mid and posterior left vocal cord	0.8	Vocal process granuloma	Minute CIS	AE1/AE3 and P63 in scattered cells, ALK1 negative	Aberrant positive	Alive and well at 68 months
13	64/M	Right ari-epiglottic fold	2.5	Carcinoma	Small foci of invasive SCC	AE1/AE3, CK 5/6, p40, p63 + in the SCC foci	Aberrant positive	Local recurrence at 14 months, treated with total laryngectomy followed by radiotherapy
14	61/M	Anterior right vocal cord	1.5	Laryngeal polyp	Minute CIS	AE1/AE3 and P63 in scattered cells, p40 negative	Aberrant positive	Alive and well at 12 months
15	79/M	Right vocal cord	1.5	Laryngeal polyp	Foci of CIS	AE1/AE3, CK5/6, p40, p63 and ALK1 negative	Aberrant positive	NA
16	80/M	Margin of the tongue	1	Pyogenic granuloma	Foci of invasive SCC	AE1/AE3, CK5/6, p40 and p63 positive	Aberrant positive	Alive and well at 9 months
17	82/F	Vocal cord	0.8	Granulation tissue polyp	Severe dysplasia	AE1/AE3, CK5/6, p63 and ALK1 negative	Aberrant negative	Recent case

Abbreviations M: Male, F: Female, NA: Not available, CIS: Carcinoma In Situ, SIL: Squamous Intraepithelial Lesion, SCC: Squamous Cell Carcinoma

**Fig. 1:**
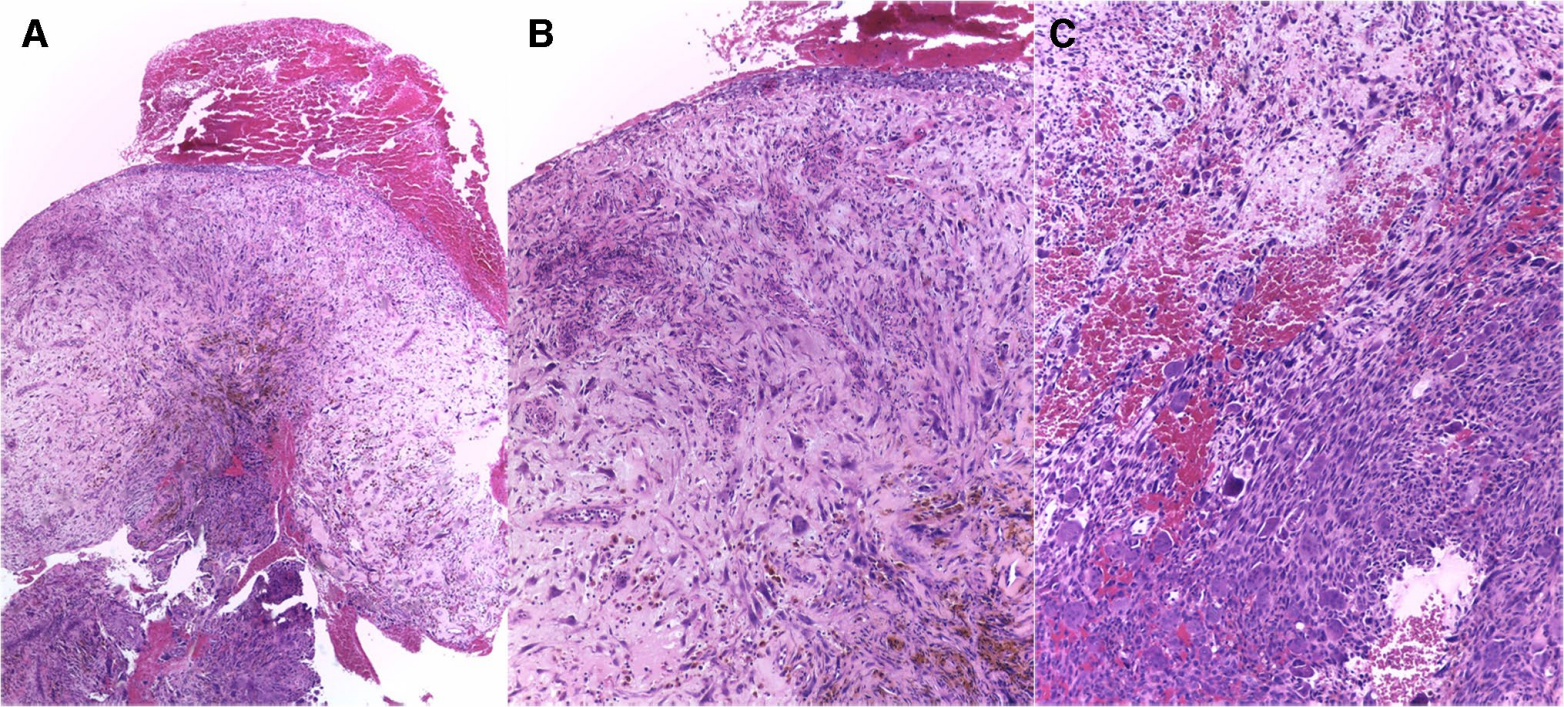
Polypoid granulation tissue-like spindle cell squamous cell carcinoma of the larynx, with ulcerated surface (**A**). The lesion consists of atypical spindle cells scattered within collagenous edematous stroma (**B**) with hemosiderin deposition. In the central portion of the lesion atypical neoplastic cells are densely packed in fascicles (**C**)

**Fig. 2:**
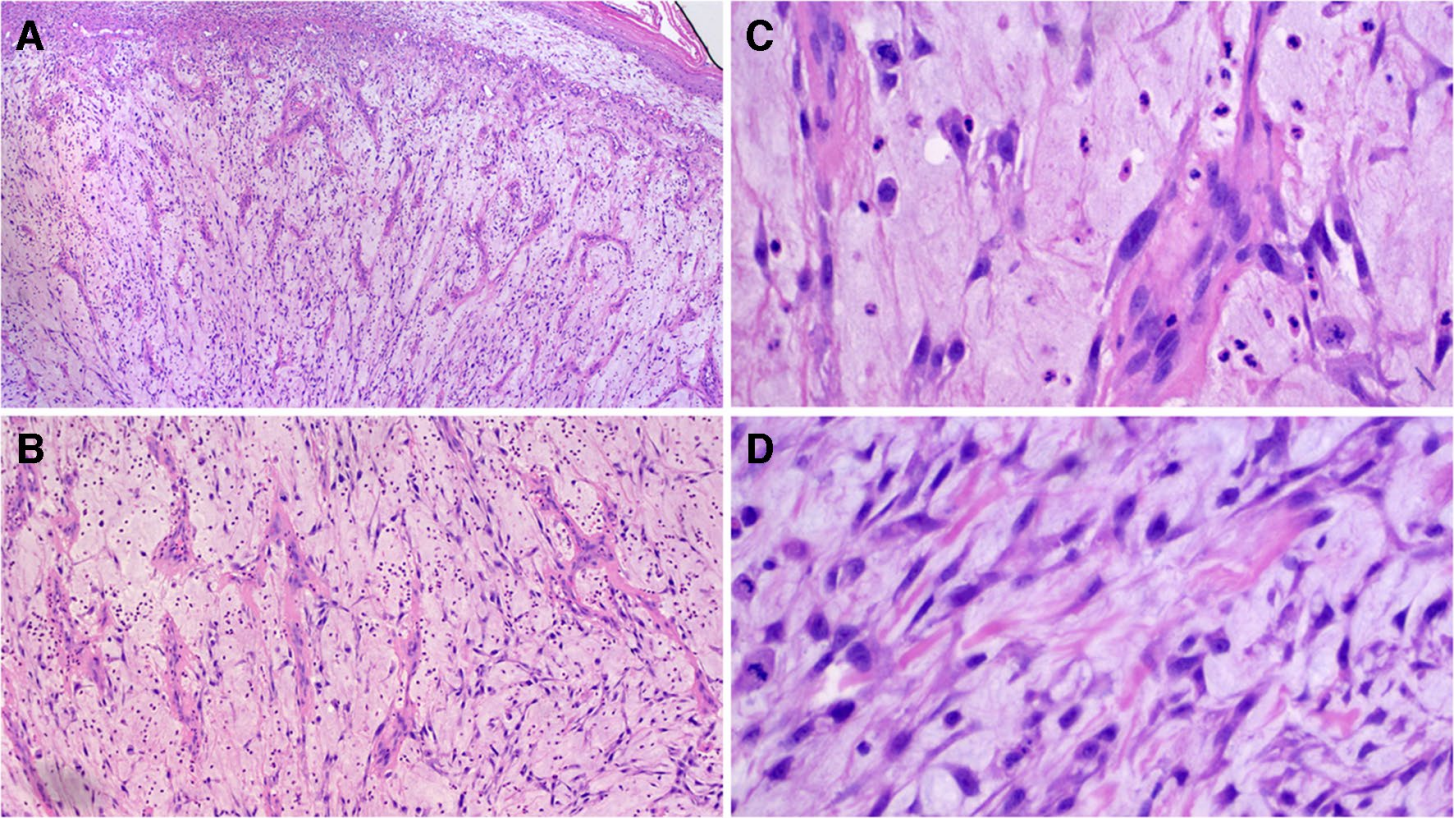
Polypoid granulation tissue-like spindle cell squamous cell carcinoma. In this example, the tumor is less cellular, and arborizing capillaries are present in the background (**A**). Neoplastic cells are interspersed within a chronic inflammatory infiltrate with edema (**B**). At high power atypical spindle cells with hyperchromatic nucleus and atypical mitotic figures are readily identified (**C**–**D**)

**Fig. 3:**
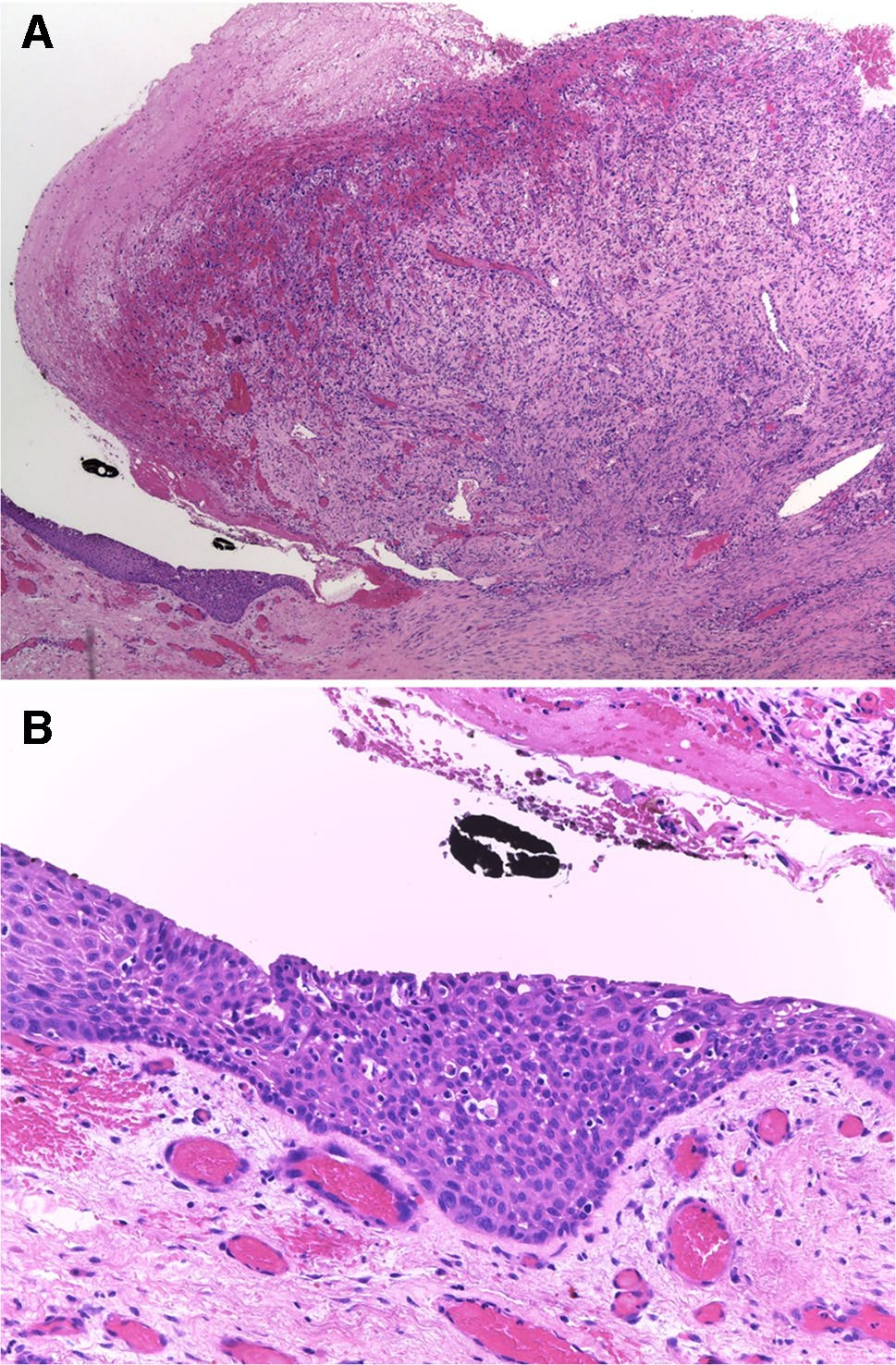
Polypoid granulation tissue-like spindle cell squamous cell carcinoma of the vocal cord (**A**). The surface epithelium is ulcerated, but high-grade dysplasia of the surface squamous epithelium at the base of the lesion is present (**B**)

**Fig. 4:**
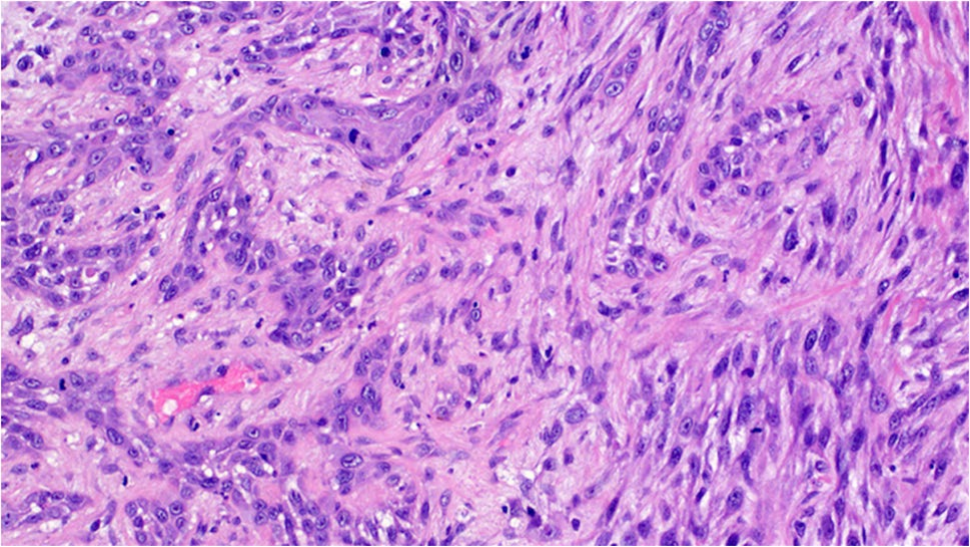
Small foci of invasive squamous cell carcinoma were identified in a few cases

Histologically, all lesions consisted of an ulcerated polypoid proliferation of moderately to markedly atypical spindle cells (Figs. 1, 2, and 3). These were either loosely distributed within the lesion (Figs. 1 and 2) or coalesced to form fascicles, mainly in the deep portion of the tumor (Fig. 1). In one case, large epithelioid cells were also present intermixed with the spindle cells. A minor component of conventional invasive (Fig. 4) or in situ squamous carcinoma (Fig. 3) was identified in five and four cases, respectively. Two further lesions of the larynx presented areas of low-grade epithelial dysplasia in the adjacent surface epithelium.

All tumors were associated with a marked inflammatory infiltrate, composed of lymphocytes, plasma cells, neutrophils, and macrophages with accompanying capillary vessels (Figs. 1, 2, and 3). Erythrocytes were interspersed within the lesion, and focal deposits of hemosiderin pigment were detected. Overall, this imparted a granulation-tissue-like appearance to the tumor. Similarity to reactive granulation tissue was most striking in the most superficial part of the lesion close to the ulceration. Distribution of the neoplastic cells in those paucicellular cases was irregular throughout the lesion and no well-organized gradual transition from above to deeper part as seen in reactive lesions was noted.

**Fig. 5:**
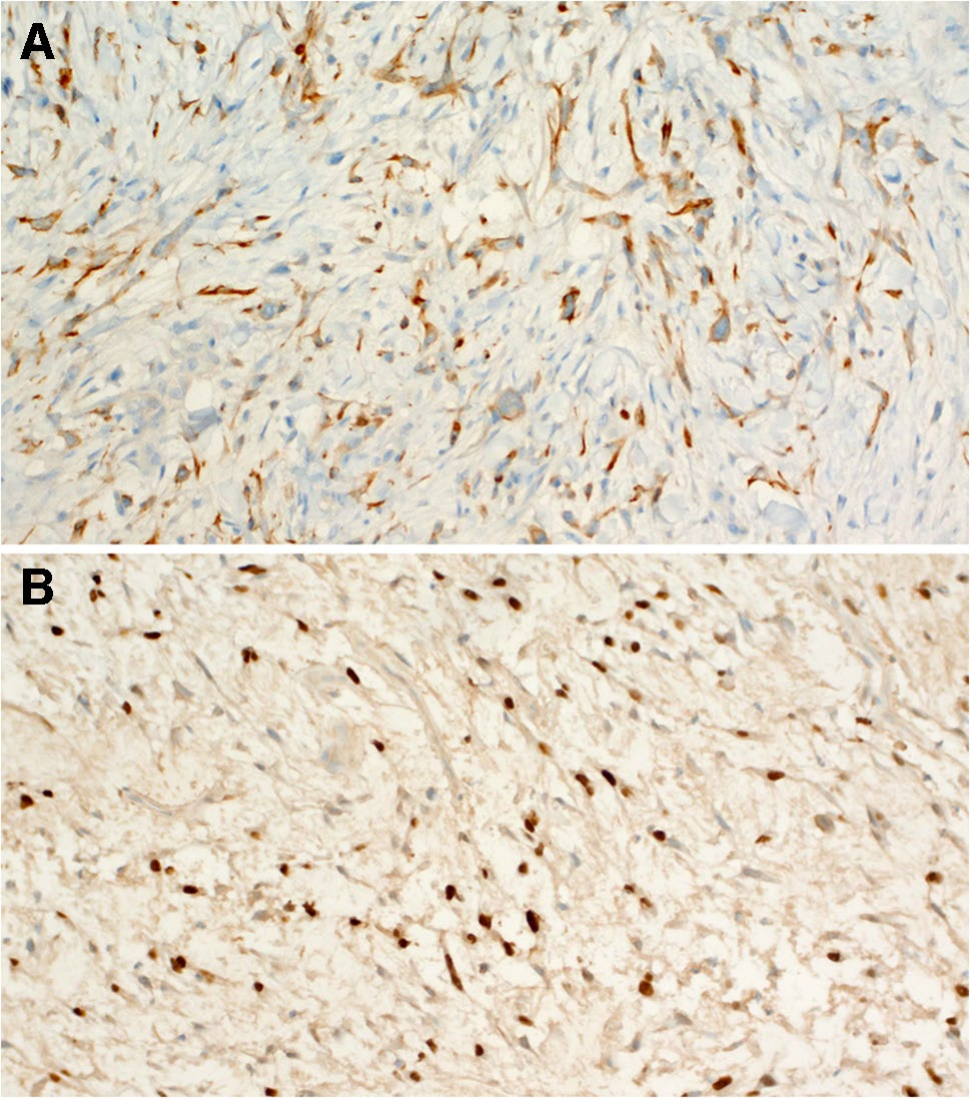
Positive immunostaining for cytokeratin CAM 5.2. (**A**). Diffuse aberrant nuclear positivity for p53 (**B**)

The results of the immunohistochemical studies are summarized in Table 1 and illustrated in Fig. 5. At least one cytokeratin cocktail was positive in 13 cases. The staining was limited to few neoplastic cells in most cases. Positivity for p63, p40, and cytokeratins 5/6 was detected only in the conventional squamous cell carcinoma component, when present. Smooth muscle actin, desmin, and ALK1 were always negative, whereas all cases demonstrated an aberrant expression of p53 indicative of *TP53* gene mutations, consisting either in diffuse nuclear staining (13 cases) or in absent (null) staining (4 cases).

All five specimens of laryngeal granulation tissue were negative for the cytokeratins tested and for ALK1, whereas smooth muscle actin and desmin were variably expressed. Nuclear p53 positivity (weak to moderate) was present in scattered epithelial, endothelial, and inflammatory cells.

## Discussion

SC-SCC is a rare variant that represents approximately 1–2% of all SCCs [6]. Most cases present as polypoid and pedunculated lesions, and, according to our study, in a significant proportion of cases, they may have a histologic appearance that closely resembles granulation tissue, thus requiring careful differential diagnosis.

In the larynx, benign lesions with granulation tissue appearance are relatively common and are altogether designated as vocal cord granulomas [9]. These lesions are found in association with gastroesophageal reflux, intubation trauma, and vocal abuse [9]. Vocal cord granuloma may affect one or both vocal cords, and in most cases, it is located in the posterior portion at the vocal process of the arytenoid, and less frequently in the middle third or the anterior portion [9]. In the present series, SC-SCC showed a different localization, with lesions mainly involving the anterior and mid vocal cord, while the vocal process of the arytenoid was never involved, and no case showed bilateral involvement. Histologically, vocal process granuloma is a polypoid lesion with a hyperplastic or ulcerated epithelial surface. The core of the lesion is represented by granulation tissue with acute and chronic inflammatory cells, abundant capillary vessels, and fibrotic changes, that become predominant with the aging of the lesion [10–12]. The distinction from SC-SCC may be difficult, and it is based on the absence of squamous dysplasia or carcinoma in situ of the overlying epithelium, as well as on the absence of nests of invasive conventional SCC or atypical cells within the core of the lesion.

In the tongue, which is the second most frequent site affected by SC-SCC in our series, the initial clinical impression was granulation tissue or lobular capillary hemangioma (pyogenic granuloma). The latter presents as a polypoid lesion with epithelial collarette and often ulcerated surface, and lobular arrangement of the proliferating vessels. Each lobule presents a central large vein encircled by several capillaries. Cellularity is often high, and mitotic activity may be brisk, but atypia is absent both in the residual surface epithelium and within the lesion.

SC-SCC with granulation tissue-like appearance must also be distinguished from inflammatory myofibroblastic tumor (IMT). This is a polypoid proliferation of fibroblasts and myofibroblasts accompanied by an inflammatory infiltrate of plasma cells, lymphocytes, and eosinophils, that may involve different mucosal sites in the head and neck. The differential diagnosis with SC-SCC is based on the absence of invasive or in situ SCC component. The immunohistochemical profile may show some overlap with that of SCC, including positivity for cytokeratins, actins, and desmin. However, IMT is also positive for ALK1 [13, 14] while in the present study all tested SC-SCC were negative. In addition, IMT harbors *ALK* gene rearrangements in 50–70% of cases, that may be useful in the differential diagnosis [15]. In recent studies, genuine IMTs of the head and neck are virtually all ALK-positive and/ or rearranged [16].

The presence of a prominent vascular component associated with atypical cells in granulation tissue-like SC-SCC brings into the differential diagnosis the possibility of a malignant vascular neoplasm, mainly Kaposi sarcoma and angiosarcomas. Involvement of head and neck mucosal sites is rare in Kaposi sarcoma and is almost exclusively observed in HIV patients [17–19]. Immunohistochemical positivity for HHV8 is the key diagnostic feature. Angiosarcoma is rare at mucosal sites of the head and neck and involves more frequently the oral cavity and the sinonasal tract, while the larynx is only exceptionally involved, often after previous radiotherapy [20–23]. The distinctive features include absence of carcinoma (in situ or invasive) and presence of tortuous and anastomosing vascular channels lined by atypical endothelial cells, which are positive for CD34, CD31, and ERG.

In this diagnostic setting, immunohistochemistry may be helpful, but it should be considered that in SC-SCC cytokeratin expression is usually decreased with the loss of epithelial differentiation, and in some cases cytokeratin expression may be lost entirely [6, 7]. Among cytokeratin markers, CK18 proved most valuable in “keratin-shy” cases [6]. Antibodies to p63 and p40 are widely used in the identification of poorly differentiated SCC and sarcomatoid carcinomas in a variety of organs, including head and neck sites [24, 25]. P53 positivity and gene mutations have been reported in SC-SCC in both the epithelial and spindle cell components [26]. In our series we observed aberrant immunohistochemical expression of TP53 in all the cases, whereas all benign lesions (granulation tissue polyps) revealed regular expression. Although the sensitivity and specificity of p53 immunostaining in this context must be further investigated, our study indicates that p53 aberrant expression could be a strong support in the diagnosis of granulation-tissue like SC-SCC, helping in the distinction from benign mimickers.

In summary, we describe the clinicopathologic features of a series of 17 cases of head and neck SC-SCC with granulation tissue-like appearance. This histologic pattern may be explained by the presence of ulcer of the surface epithelium that induces marked inflammation and proliferation of capillaries. Such changes may be so intense and diffuse to obscure the underlying neoplastic component, and thus careful differential diagnosis with benign lesions, including granulomas and vascular lesions, is mandatory. These tumors are often initially misdiagnosed, and in case of recurrence of a granulation tissue polyp of the upper aerodigestive tract mucosa, the possibility of a granulation tissue-like SC-SCC should be considered. Immunohistochemistry often cannot identify epithelial differentiation, but this should not preclude the diagnosis of SC-SCC, especially if there is evidence of dysplasia or carcinoma in situ of the adjacent surface epithelium. Evaluation of p53 immunohistochemical expression may be a most useful adjunct to distinguish these carcinomas from benign mimickers.

## Data Availability

Data supporting the findings of this study are available within the article.
